# Construction autophagy-related prognostic risk signature to facilitate survival prediction, individual treatment and biomarker excavation of epithelial ovarian cancer patients

**DOI:** 10.1186/s13048-021-00791-3

**Published:** 2021-03-06

**Authors:** Hongjun Fei, Songchang Chen, Chenming Xu

**Affiliations:** 1 Department of Reproductive Genetics, International Peace Maternity and Child Health Hospital, Shanghai Key Laboratory of Embryo Original Diseases, Shanghai Municipal Key Clinical Specialty, Shanghai Jiao Tong University School of Medicine, No.910, Hengshan Road, Shanghai, 200030 People’s Republic of China; 2 Obstetrics and Gynecology Hospital of Fudan University, Shanghai, 200011 China

**Keywords:** Epithelial ovarian Cancer, Autophagy-related genes, Prognostic risk model, Targeted therapeutic intervention, Survival prediction

## Abstract

**Background:**

Existing clinical methods for prognosis evaluating for Epithelial Ovarian Cancer (EOC) patients had defects of invasive, unsystematic and subjective and little data are available for individualizing treatment, therefore, to identify potential prognostic markers and new therapeutic targets for EOC is urgently required.

**Results:**

Expression of 232 autophagy-related genes (ARGs) in 354 EOC and 56 human ovarian surface epithelial specimens from 7 independent laboratories were analyzed, 31 mRNAs were identified as DEARGs. We did functional and pathway enrichment analysis and constructed protein–protein interaction network for all DEARGs. To screen out candidate DEARGs related to EOC patients’ survival and construct an autophagy-related prognostic risk signature, univariate and multivariate Cox proportional hazards models were established separately. Finally, 5 optimal independent prognostic DEARGs (*PEX3*, *DNAJB9*, *RB1*, *HSP90AB1* and *CXCR4*) were confirmed and the autophagy-related risk model was established by the 5 prognostic DEARGs. The accuracy and robustness of the prognostic risk model for survival prediction were evaluated and verified by analyzing the correlation between EOC patients’ survival status, clinicopathological features and risk scores.

**Conclusions:**

The autophagy-related prognostic risk model can be independently used to predict overall survival in EOC patients, it can also potentially assist in individualizing treatment and biomarker development.

## Introduction

Ovarian cancer (OC) has the highest morbidity and mortality in the female genital tract [1]. It is the fifth most frequent cause of cancer death in women in the United States in 2020 which results in the death of 5% women with cancer, and 5-year survival rates are 39–48% of all the women diagnosed with ovarian cancer [2, 3]. Epithelial ovarian cancer (EOC) is the most common type of OC which accounts for almost 90% of all ovarian cancers [4, 5]. It generally presents at an advanced stage in over 70% of patients contributing to a high death rate, where the long term survival rate (10 years) is estimated at 15–30% [6, 7]. The prognosis of EOC is associated with many factors such as histological type, pathological stage, age, early recognition, the volume of ascites and so on [8, 9], existing clinical methods for prognosis evaluation still had defects of invasive, unsystematic and subjective. It is necessary to refine the prognostic model of EOC and establish a more accurate method to managing this high-mortality disease.

Establishing prognosis models for EOC patients is an important part of risk evaluation and treatment, can also greatly assists in biomarker development [10]. From clinically applicability, prognosis model is a handy tool to estimate overall survival and risk of recurrence, and can potentially help in individualizing treatment for patients [11]; From research development, it can contribute to identify subgroups of patients with unfavorable prognosis and promote us to explore alternative treatment strategies for these patients, and provide an idea for targeting therapy [12]. The present study focuses on constructing a prognosis model for EOC by molecular typing methods using large databases.

Autophagy is a homeostatic mechanism that can maintain cell survival by recycling organelles and macromolecules [13]. Various genes named because of participating in autophagy, they were called Autophagy-Related Genes (ARGs) [14]. Accumulating data suggest that autophagy dysregulation in EOC cells caused dormancy and chemo−/radio-therapy resistance, and the process involving proteins (mainly ARGs encoding proteins) are being considered as anticancer molecular therapeutic targets [15–18]. However, no prior study used the large-scale expression patterns for assessing the autophagy effect on EOC prognosis. So, this study makes use of ARGs to construct the prognostic risk signature of EOC. Through this novel prognostic risk model, we expect to shed light on prognosis evaluation and targeted treatment of EOC.

## Materials and methods

### Data acquisition

The gene expression profiling data sets (ID: GSE14407, GSE6008, GSE14001, GSE26712, GSE29450, GSE38666, GSE105437) were obtained from Gene Expression Omnibus database (https://www.ncbi.nlm.nih.gov/geo/). The brief information of 7 GEO datasets [containing 410 human ovarian surface epithelial (HOSE) and epithelial ovarian cancer (EOC) specimens] from 7 independent laboratories was extracted and listed in Table 1. The RNA-seq data and the corresponding clinical data of 379 EOC patients were downloaded from the TCGA database (https://portal.gdc.cancer.gov/). We downloaded 232 genes identified so far to be involved in autophagy from the Human Autophagy Database (HADb).

**Table 1:** Characteristic of microarray data used to do difference analysis

Expression profiling array (HOSE & EOC)	Platforms	GEO accession	Samples
Genome	GPL570	GSE14407	12HOSE; 12EOC
		GSE14001	3HOSE; 20EOC
		GSE29450	10HOSE; 10EOC
		GSE38666	12HOSE; 18EOC
		GSE105437	5HOSE; 10EOC
Genome	GPL96	GSE6008	4HOSE; 99EOC
		GSE26712	10HOSE; 185EOC

### Differentially expressed autophagy-related genes (DEARGs) screening

Data preprocessing was performed before difference analysis for 7 independent GEO datasets. We did batch normalization for all expression profiling data through ComBat algorithm in R to eliminate the systematic variations among different studies. The differentially expressed autophagy-related genes (DEARGs) between 354 EOC tissues and 56 HOSE tissues were screened out by the Wilcoxon signed-rank test. The cutoff criteria were adjusted *p*-value < 0.05 and |log_2_FoldChange| (|log_2_FC|) > 1.

### Protein–protein interaction (PPI) network construction for all DEARGs

PPI analysis is a protein correlation analysis that can effectively reveal the molecular mechanisms of crucial cellular activities in carcinogenesis. It is constructed based on the STRING database (https://string-db.org/). The PPI network was constructed for all DEARGs and visualized with the cut-off criterion of interaction score > 0.4. To visualized the PPI network and highlighted the hub genes, we make use of the Cytoscape software to perform deeply biological network analysis.

### Functional and pathway enrichment analysis for all DEARGs

Gene Ontology (GO) analysis can annotate characteristics of a set of genes, such as involved cellular components (CC), molecular functions (MF) and biological processes (BP). Kyoto Encyclopedia of Genes and Genomes (KEGG) analysis was used to reveal the involved pathways of all DEARGs. These analyses were done by clusterProfiler package of R with *p* value < 0.05 as a strict cutoff.

### Identify the prognostic DEARGs

To identify DEARGs whose expression profiles had a significant correlation with the overall survival (OS) of patients with EOC, we performed the univariate Cox regression model. The DEARGs with the threshold of *P* < 0.05 were regarded as candidate genes related to EOC patients’ survival.

### Construction of OS risk prognostic model and risk score calculation

The survival-related DEARGs screened out through the univariate Cox proportional hazards model were incorporated into a multivariate Cox regression analysis to weeded out the DEARGs which might not be an independent index in prognosis monitoring. Then, we can obtain a set of optimal prognostic DEARGs, and establish a risk score model using these genes.

We can get the risk score for each EOC patient through the followed formula,

$$ \mathrm{the}\ \mathrm{risk}\ \mathrm{score}=\sum \limits_{\mathrm{i}=1,2,\dots, \mathrm{n}}\mathrm{regression}\ \mathrm{coeffiecient}\left(\mathrm{genei}\right)\times \mathrm{expression}\ \mathrm{value}\ \mathrm{of}\ \left(\mathrm{genei}\right) $$

The risk score was calculated using the gene expression value multiplied by the regression coefficients of each individual gene. The genes in the formula mean the independent prognostic DEARGs we screened before. The regression coefficients were obtained from the multivariate Cox regression model and stand for the relative weight of selected genes. It is a measure of prognostic risk for EOC patients. With the median risk score as the cutoff value, patients were divided into low-risk group and high-risk group. A high-risk score means a poor prognosis.

### Evaluation of OS risk prognostic model

We draw the survival curves by Kaplan–Meier plotter and assessed the difference in the survival status between the high-risk group and low-risk groups to verify the validity and robustness of the OS risk prognostic model. Then, the relationship between risk score and survival status of EOC patients was visualized. We studied genetic alterations of 5 independent prognostic DEARGs (*PEX3*, *DNAJB9*, *RB1*, *HSP90AB1* and *CXCR4*) in an Ovarian Cystadenocarcinoma case set using an open-access tool cBioPortal (http://www.cbioportal.org).

What’s more, we performed Cox proportional hazard regression to evaluate whether the OS risk prognostic model constructed with DEARGs could independent of other clinicopathological features as a predictor of OS for EOC patients. The correlation between the risk score and clinical traits was analyzed by univariate and multivariate Cox regression methods. Age, pathological stage and histological grade were used as candidate clinicopathological risk factors. We want to explore whether clinicopathological risk factors and risk scores calculated based on prognostic DEARGs can all be indicators in prognosis monitoring for EOC patients, and to confirm whether the risk score could be an independent prognosis factor.

## Results

### Identification of differentially expressed autophagy-related genes (DEARGs)

**Fig. 1:**
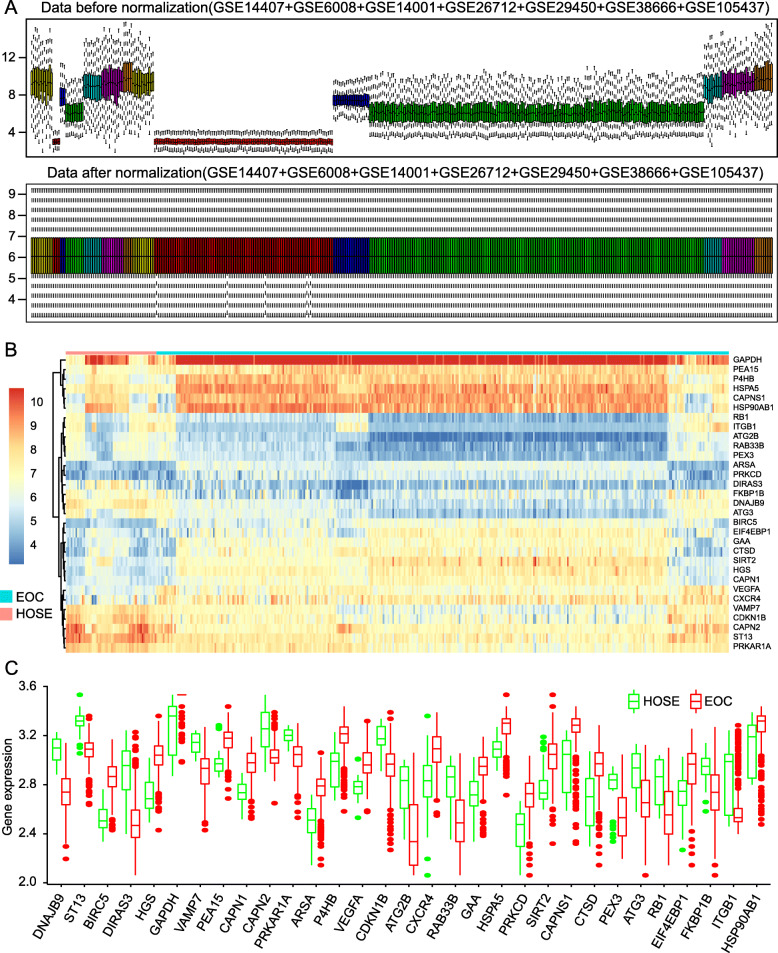
Differentially expressed autophagy-related genes (DEARGs) screened from 354 EOC tissues and 56 HOSE tissues. **a** The expression values of all genes before and after normalization were displayed by box Figs. X-axis represents 410 tissues samples from GSE14407, GSE6008, GSE14001, GSE26712, GSE29450, GSE38666, and GSE105437 datasets. The 7 groups on the left were HOSE tissues, and the right were EOC tissues. The same color columns represent samples that came from the same GEO datasets. **b** Heatmap of the expression levels of 31 DEARGs in EOC. The depth of blue and red color represents the intensity of the expression level of DEARGs. EOC, Epithelial Ovarian Cancer. **c** Visualization of expression patterns of 31 DEARGs. Red box plots represent EOC samples and green represent HOSE samples

The expression values of 13,045 genes in 410 samples (354 EOC and 56 HOSE specimens) were normalized with R package and showed in Fig. 1**a**. Horizontal axis and vertical axis represents 410 samples and all gene expression value, respectively. After batch normalization, the median of expression value of samples is almost the same, proved normalized data were qualified. The expression values of 232 ARGs were extracted. Considering adjust *p*-value < 0.05 and |log_2_FC| > 1 as the filter criteria, 31 DEARGs (14 downregulated ARGs and 17 upregulated ARGs) were obtained. We list the details such as log_2_FoldChange and statistical significance of all DEARGs in Table 2. In Fig. 1**b**, the fold change patterns of 31 DEARGs were showed in a heat map. The expression of 31 DEARGs between EOC tissues and HOSE tissues was visualized by scatter plots (Fig. 1**c**).

**Table 2:** All DEARGs, screened between human ovarian surface epithelia (HOSE) tissues and epithelial ovarian cancer (EOC) tissues with criteria of adjust-*p*-Value < 0.05 and | log_2_FoldChange| > 1

Gene	Log_2_FC	*p*-Value	adjust-*p*-Value
DNAJB9	−1.774	7.49E-49	1.44E-46
ST13	−1.484	2.45E-46	2.36E-44
BIRC5	1.474	5.62E-45	3.61E-43
DIRAS3	−1.867	2.79E-39	1.34E-37
HGS	1.599	9.39E-39	3.62E-37
GAPDH	1.628	1.77E-35	4.27E-34
VAMP7	−1.290	3.20E-30	6.17E-29
PEA15	1.065	1.02E-29	1.60E-28
CAPN1	1.165	1.08E-29	1.60E-28
CAPN2	−1.263	6.24E-29	8.60E-28
PRKAR1A	−1.055	1.38E-27	1.67E-26
ARSA	1.077	6.94E-26	6.69E-25
P4HB	1.387	1.09E-25	9.98E-25
VEGFA	1.027	3.12E-25	2.74E-24
CDKN1B	−1.338	1.64E-24	1.32E-23
ATG2B	−1.581	3.71E-24	2.75E-23
CXCR4	1.353	2.39E-23	1.59E-22
RAB33B	−1.291	6.16E-23	3.72E-22
GAA	1.086	4.38E-22	2.49E-21
HSPA5	1.155	6.10E-22	3.27E-21
PRKCD	1.104	1.42E-21	7.22E-21
SIRT2	1.210	5.31E-19	2.23E-18
CAPNS1	1.574	6.48E-19	2.66E-18
CTSD	1.250	1.68E-18	6.47E-18
PEX3	−1.012	7.33E-18	2.57E-17
ATG3	−1.069	3.08E-16	9.59E-16
RB1	−1.121	3.90E-15	1.12E-14
EIF4EBP1	1.002	2.87E-13	7.39E-13
FKBP1B	−1.039	1.63E-12	3.99E-12
ITGB1	−1.107	2.73E-12	6.42E-12
HSP90AB1	1.028	4.50E-09	8.69E-09

### PPI network construction and functional annotation of DEARGs

**Fig. 2:**
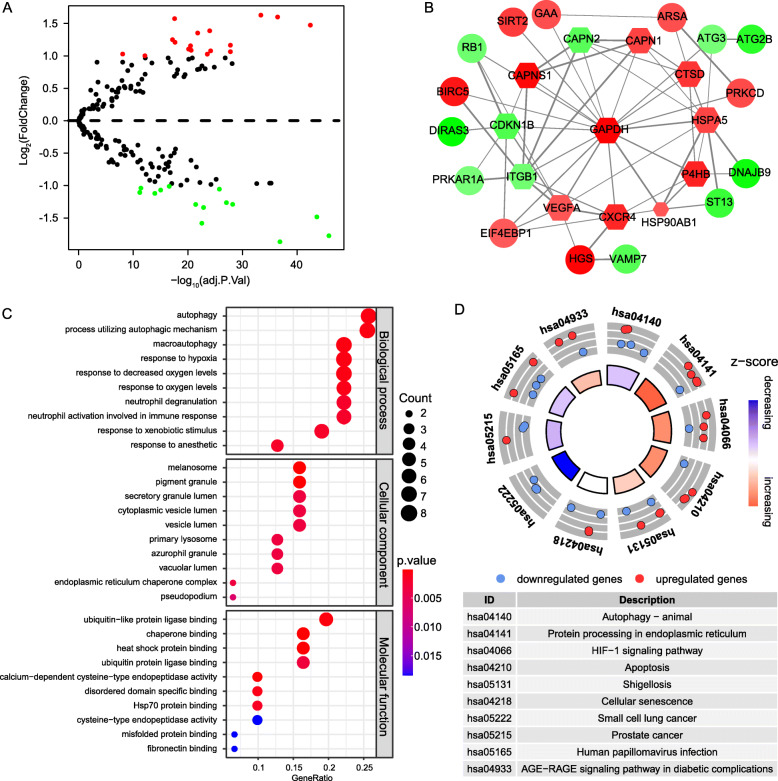
PPI network construction and functional enrichment analyses of DEARGs. **a** Volcano plot of 242 autophagy-related genes (ARGs). The red spots and green spots represent up-regulated genes and down-regulated genes which |Log_2_FoldChange| > 1.0. **b** PPI network of 31 DEARGs. The color of nodes is associated with log_2_FoldChange, red nodes denote up-regulated DEARGs and green nodes denote down-regulated DEARGs. The depth of red and green color represents the value size of log_2_FoldChange. The width of the edge is associated with a combined score of protein interaction. The size of nodes is inversely related to *p*-value. Diamond nodes are hub genes whose interactive protein more than 5. **c** Gene Ontology analysis of 31 DEARGs. **d** Kyoto Encyclopedia of Genes and Genomes analyses of 31 DEARGs showed the involved signaling pathways

We displayed the distribution of all DEARGs with a volcano plot (Fig. 2**a**). A PPI network of DEARGs was constructed with STRING database and visualized the interaction of the PPI network using Cytoscape software, there are 12 hub genes with interaction degree > 5 showed as a diamond and arranged in a circle (Fig. 2**b**). GO analysis shows that DEARGs are enriched in several hypoxia-related biological processes (BP), including autophagy, macroautophagy, response to hypoxia and response to oxygen levels. Regarding the molecular function (MF), the DEARGs played vital parts in some functions, such as ubiquitin-like protein ligase binding, chaperone binding and heat shock protein binding. In terms of the cellular components (CC), the proteins encoded by the DEARGs are components of melanosome, pigment granule and secretory granule lumen (Fig. 2**c**). KEGG analysis showed the main pathways that DEARGs involved containing autophagy, and protein processing in the endoplasmic reticulum (Fig. 2**d**).

### Establishment of autophagy-related signature

**Fig. 3:**
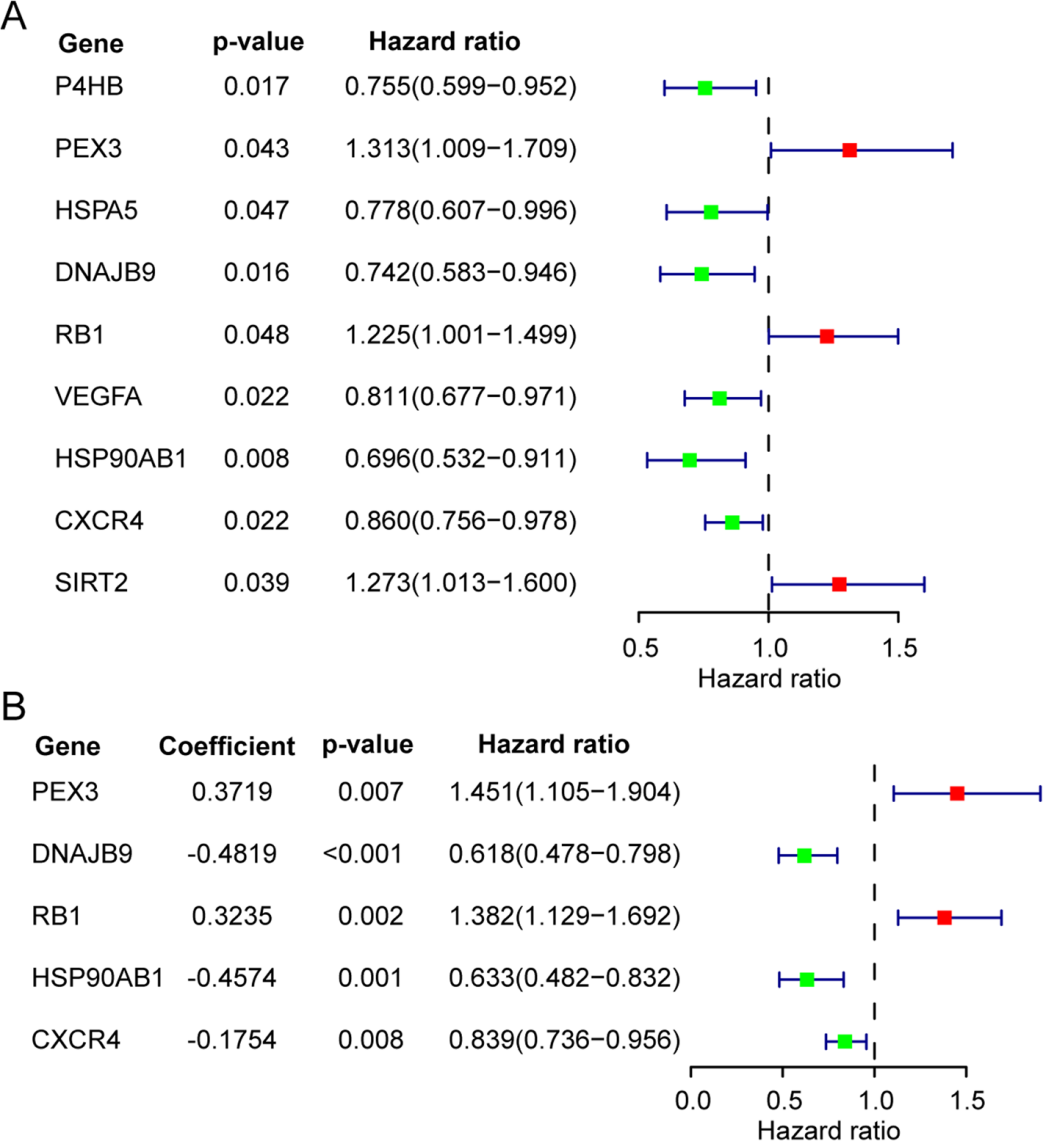
Identify differentially expressed autophagy genes which related to the prognosis of EOC and construct a prognostic risk model. Univariate and multivariate regression analyses evaluated the relationship between expression patterns of DEARGs and overall survival (OS) in Epithelial Ovarian Cancer (EOC) patients. **a** Significance and Hazard ratio values of 9 prognostic DEARGs obtained from univariate Cox regression analysis. **b** Regression coefficients, significance and hazard ratios values of 5 independent prognostic DEARGs obtained from multivariate Cox regression

Altogether RNA-seq and clinical data of 379 EOC tissue specimens were obtained from the TCGA database and 374 EOC tissue specimens contained complete clinical follow-up information were subjected to univariate Cox regression analyses to evaluate the association between 31 screened DEARGs’ expression profiles and overall survival in 374 EOC patients. The results of univariate Cox regression analyses revealed that 9 DEARGs were significantly associated with the prognosis of EOC patients (*p* < 0.05) (Fig. 3**a**). To improve the validity and robustness, 9 prognostic-related DEARGs obtained from the above univariate analysis were further subjected to the multivariate Cox regression analysis. Finally, 5 DEARGs (*PEX3*, *DNAJB9*, *RB1*, *HSP90AB1* and *CXCR4*) were screened out as optimal independent prognosis biomarkers and applied to construct an autophagy-related risk model (Fig. 3**e**). The risk score of OS for EOC patients was calculated according to the following formula: risk score = (0.3719 ✕ expression value of *PEX3*) + (− 0.4819 ✕ expression value of *DNAJB9*) + (0.3235 ✕ expression value of *RB1*) + (− 0.4574 ✕ expression value of *HSP90AB1*) + (− 0.1754 ✕ expression value of *CXCR4*). The risk scores of 374 EOC patients were calculated through the above formula, and patients were divided into high-risk (*n* = 187) and low-risk group (n = 187) with the median of the risk score as the cutoff value.

### Validation of the risk signature

**Fig. 4:**
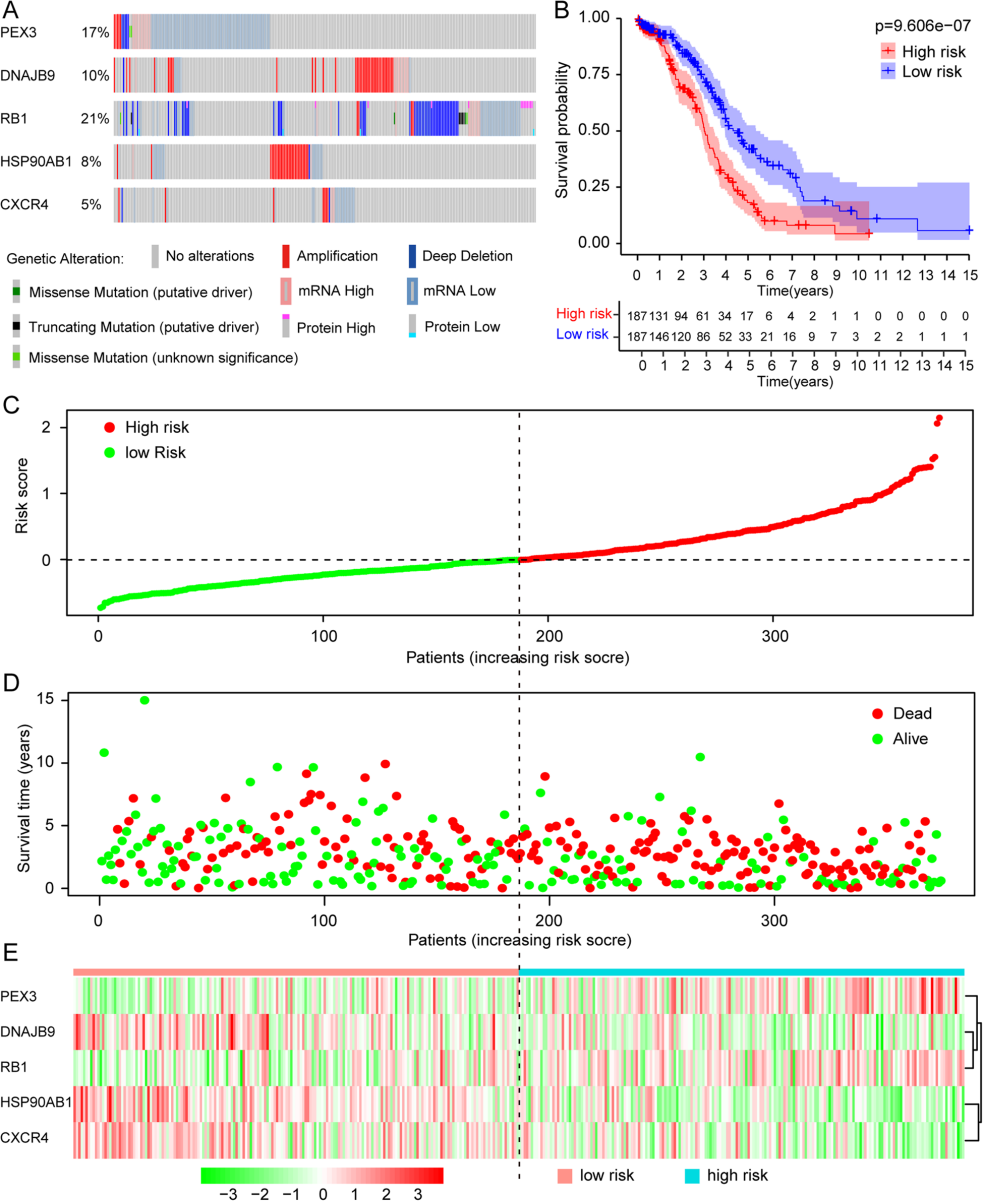
Evaluation of prognostic model. **a** Genetic alterations analysis of 5 prognostic DEARGs. **b** Overall survival for EOC patients in the low-risk and high-risk group was analyzed by the Kaplan-Meier survival curve. **c** Distribution of the risk scores of EOC patients. **d** The scatter plot shows the survival status of EOC patients in different risk groups, the red plot represents non-survivors, and the green means survivors. **e** The expression profiles of the prognostic DEARGs which used to construct a prognostic model in the high-risk (blue) and low-risk (pink) group

The OncoPrint in cBioPortal is a compact and efficient graphical summary of genomic alterations in tumor specimens. According to previous results of DEARGs screening and autophagy-related signature establishment, the risk score calculated based on the expression of *PEX3*, *DNAJB9*, *RB1*, *HSP90AB1* and *CXCR4* can be a predictor of OS for EOC patients independent of other clinicopathological parameters. We analyzed and visualized genomic alterations of 5 prognostic DEARGs by cBioPortal in ovary carcinoma cases **(**Fig. 4**a)**. Kaplan-Meier plot was drawn to compare the OS difference between high-risk and low-risk group. EOC patients in the low-risk group had obviously better survival outcomes than the high-risk group (*p* = 9.606E-07) illustrated that the risk score based on the autophagy-related risk model correlated with EOC patients’ prognosis (Fig. 4**b**). Figure.4**c** visualized the risk scores from low to high. The mortality of EOC patients increased as the risk score increased (Fig. 4**d**). The heatmap in Fig. 4**e** displayed the expression patterns of 5 prognostic DEARGs in different risk groups.

### Clinical utility of prognostic signature

**Fig. 5:**
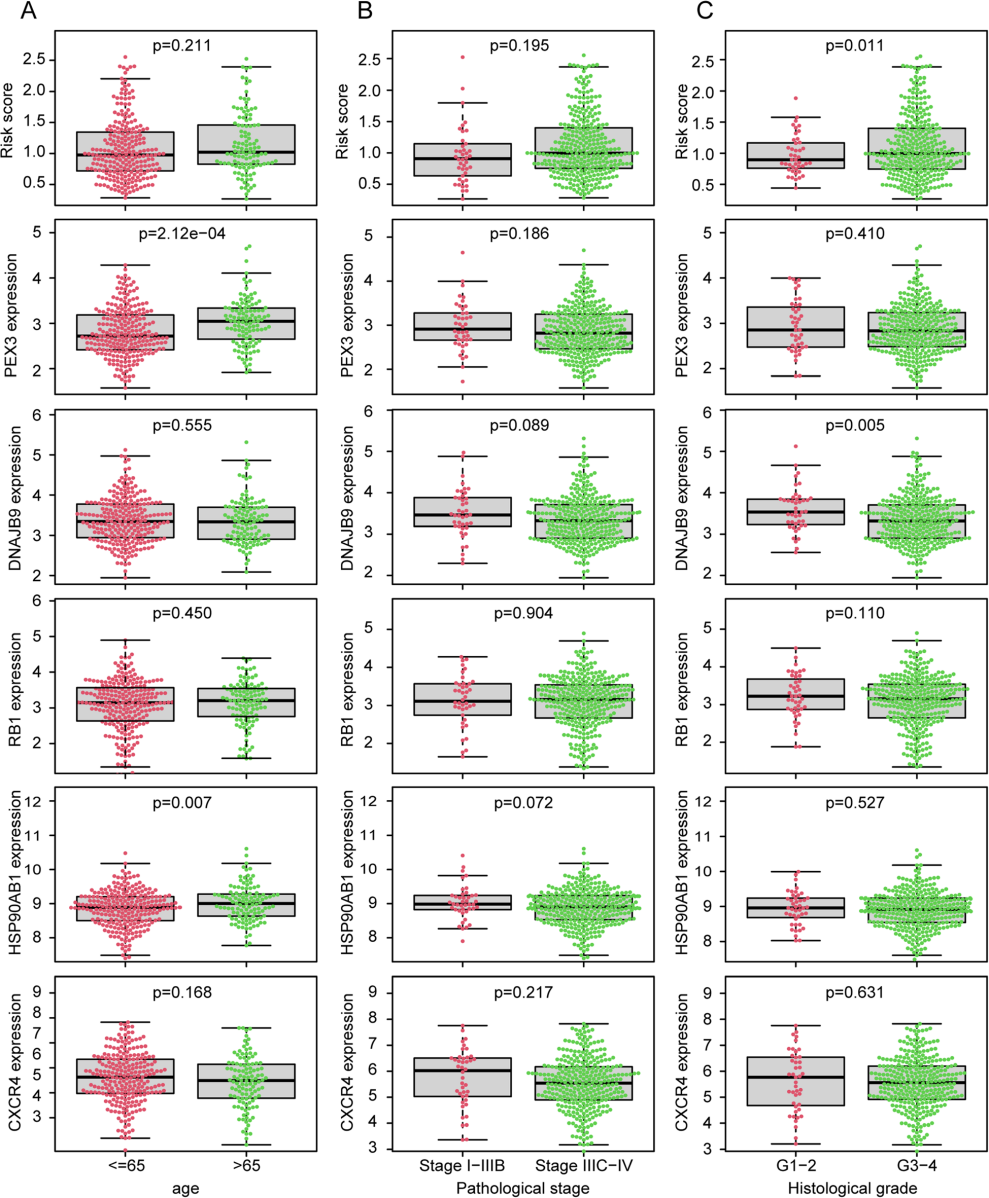
Clinical correlations among the risk score, prognostic DEARGs and clinicopathological variables. **a** Age. **b** Pathological stage. **c**. Histological grade. It showed the clinicopathological significance of the OS-related prognostic model constructed based on autophagy-related signature in EOC

The univariate and multivariate Cox proportional hazard regression analyses were performed to determine the correlation between the risk score and clinicopathological features, and to further evaluated whether the autophagy-related risk prognostic model constructed with DEARGs could independent of other clinicopathological features as a predictor of OS for EOC patients. There is no difference of risk scores between age > 65 and age ≤ 65 (*p* = 0.211) (Fig. 5**a**) or pathological stage IIIC-IV and pathological stage I-IIIB (*p* = 0.195) (Fig. 5**b**), although elder patients seemed had a higher risk score than younger patients and high pathological stage patients seemed had a higher risk score than low pathological stage patients. Risk score was higher in histological grade G3–4 than in G1–2 (*p* = 0.011) (Fig. 5**c**). Obviously, the majority of EOC patients are diagnosed at a late stage, so most patients are diagnosed with pathological stage III or histological grade 3–4. The association between the expression level of 5 prognostic DEARGs which used to construct the risk model and clinical pathological parameters of EOC are shown in Fig. 5**a, b, c**.

In Table 3, univariate and multivariate Cox regression analysis showed that age and risk score was significantly correlated with OS of EOC, risk score can be an independent factor for the OS of EOC. These results confirmed that the autophagy-related prognostic signature can be an independent indicator for prognosis monitoring for EOC patients.

**Table 3:** Univariate and multivariate cox regression analyses of riskscore and clinicopathologic features in the TCGA group EOC patients

Variables	Univariate analysis		Multivariate analysis	
	HR (95% CI)	*p*-Value	HR (95% CI)	*p*-Value
RiskScore	2.120(1.629–2.759)	**< 0.001**	2.107(1.609–2.760)	**< 0.001**
Age	1.020(1.006–1.033)	**0.004**	1.020(1.007–1.034)	**0.003**
Pathological Stage	1.353(0.999–1.832)	0.051	1.300(0.947–1.786)	0.105
Histological Grade	1.448(0.968–2.165)	0.071	1.244(0.828–1.868)	0.293

## Discussion

Autophagy is a eukaryotic cellular degradation and recycling process which is highly conserved [19]. Many studies had confirmed that autophagy plays a significant role in EOC [20–23]. Some pharmaceutical agents targeting autophagy have been proved effective in EOC patients [24, 25]. Up to now, histological grade and stage remain the strongest prognostic evaluation tools in EOC [26]. Make use of a prognostic model which constructed based on the specific molecule can improve the understanding of the molecular mechanism of EOC, aid the development of more specific therapies, and identify novel biomarkers [27, 28]. So, we construct autophagy-related prognostic signature with DEARGs to predict the prognosis of EOC patients.

According to our study, an autophagy-related independent prognostic signature is constructed by 5 ARGs with different coefficients, including *PEX3*, *DNAJB9*, *RB1*, *HSP90AB1* and *CXCR4*. There were only a few researches reported a relationship between the 5 ARGs and cancers, Daniela et al and Shaobo et al reported PEX3 plays an important role in Melanoma [29] and colon cancer [30] respectively. *DNAJB9* is a known negative feedback regulator of the tumor suppressor gene p53 in non-gestational choriocarcinoma [31] and can reduce chemotherapy resistance in acute myeloid leukemia [32]. Guang et al reported that *RB1* is a tumor suppressor in OC [33]. *HSP90AB1* previously known as heat shock 90-kDa protein 1, beta, its expression is relatively stable in ovarian tissues [34]. As a chemokine receptor, blocking the CXCR4/CXCL12 signal could be a potential therapy for EOC patients [35]. The results suggest that most autophagy-related independent potential prognostic markers were identified and verified in other cancers except EOC, hence identifying potential prognostic markers and new therapeutic targets for EOC patients is essential.

There are 374 EOC patients’ information with gene expression and survival data were obtained from TCGA to established an autophagy-related prognostic model, and only 364 patients with complete clinical information of age, pathological stage and histological grade. We verified the relationship between autophagy-related prognostic signature and their clinicopathological features. The 5 ARGs are not all associated with clinicopathological features of EOC patients, but the risk score calculated based on 5 prognostic DEARGs is significantly related to the histological grade of EOC patients. It is important to notice that among 364 EOC patients, only 43 patients were subjected to G1-G2, 321 patients were subjected to G3-G4. The same is only 22 patients diagnosed in stage I-II, the rest 342 patients all diagnosed in stage III-IV. Many patients diagnosed at an advanced stage disturbed the analysis for the relationship between the risk model and clinical information. Even so, we still can find a trend of risk score was higher in pathological stage IIIC-IV than in stage I-IIIB. Several assessment methods confirmed that the prognostic model can be an independent indicator for prognosis monitoring for EOC patients. Clinical information of EOC patients reminded us that early diagnosis is urgently needed for EOC patients. Our study shed light on finding the diagnosis and targeted treatment biomarkers in EOC patients.

Pathway enrichment analysis showed that 31 DEARGs were mainly involved in hypoxia or oxygen related pathways. Kalpana et al reported that hypoxia-induced carcinoma progression and metastasis, and drug resistance are serious problems for EOC treatment in clinical [36]. Edith et al proposed that OC metastasizes and recurs all in a unique hypoxia microenvironment in the abdominal cavity [37]. Hence, the affection of hypoxia to EOC patients should be evaluated and managed cautiously.

Our study developed a risk prognostic model to predict individuals’ clinical outcomes, molecular signature combined with clinical features make the model steady and credible. Our results proved that the risk model constructed by 5 DEARGs *PEX3*, *DNAJB9*, *RB1*, *HSP90AB1* and *CXCR4* is clinically practicable to evaluated prognosis for EOC patients. Besides traditional clinicopathological indicators (including pathological stage and histological grade), risk scores based on the autophagy-related genes signature could also be applied in clinical to provide a handy and better prognosis monitoring. The DEARGs can also help facilitate personalized target treatment and early screening.

## Conclusions

Our study analyzed transcriptome expression profiles of 354 EOC tissue samples and 56 HOSE samples from 7 independent laboratories and evaluated the expression pattern of 232 ARGs in the two groups. There were 17 up-regulated DEARGs and 14 down-regulated DEARGs in EOC with the filter criteria of adjusting *p*-value< 0.05 and |log_2_FC| > 1.0. From 31 DEARGs, 5 prognostic DEARGs (*PEX3*, *DNAJB9*, *RB1*, *HSP90AB1, CXCR4*) were identified to construct a risk score prediction model, the risk score was calculated with the expression values of these genes and regression coefficients. Combined validation analysis of molecular signature and clinical characteristics, the risk score prediction model can robustly estimate the OS of EOC patients. The prognosis DEARGs also provide new possibilities for EOC therapeutic intervention. Meanwhile, our study can reveal the molecular mechanisms behind EOC from a brand-new insight.

## Data Availability

The raw data supporting the conclusions of this manuscript will be made available by the authors, without undue reservation, to any qualified researcher.

## Abbreviations

ARGs: Autophagy- related genes
EOC: Epithelial ovarian cancer
DEARGs: Differentially expressed autophagy- related genes
HOSE: Human ovarian surface epithelia
GO: Gene Ontology
KEGG: Kyoto Encyclopedia of Genes and Genomes
KM plotter: Kaplan-Meier plotter
log_2_FC: log_2_Fold Change/Logarithm of Fold Change
OC: Ovarian cancer
OS: Overall survival
PPI: protein-protein interaction
TCGA: The Cancer Genome Atlas
